# Effects of Telephone Counseling Intervention by Pharmacists (TelCIP) on Medication Adherence; Results of a Cluster Randomized Trial

**DOI:** 10.3389/fphar.2016.00269

**Published:** 2016-08-30

**Authors:** Marcel J. Kooij, Eibert R. Heerdink, Liset van Dijk, Erica C. G. van Geffen, Svetlana V. Belitser, Marcel L. Bouvy

**Affiliations:** ^1^Department of Pharmacoepidemiology and Clinical Pharmacology, Utrecht UniversityUtrecht, Netherlands; ^2^Service Apotheek KoningAmsterdam, Netherlands; ^3^NIVEL (Netherlands Institute for Health Services Research)Utrecht, Netherlands; ^4^Kidney Foundation (Nierstichting)Bussum, Netherlands

**Keywords:** medication adherence, intervention, counseling, pharmaceutical care, statins, antidepressant, bisphosphonate, RAS-inhibitor

## Abstract

**Objectives:** To assess the effect of a pharmacist telephone counseling intervention on patients' medication adherence.

**Design:** Pragmatic cluster randomized controlled trial.

**Setting:** 53 Community pharmacies in The Netherlands.

**Participants:** Patients ≥18 years initiating treatment with antidepressants, bisphosphonates, Renin-Angiotensin System (RAS)-inhibitors, or statins (lipid lowering drugs). Pharmacies in arm A provided the intervention for antidepressants and bisphosphonates and usual care for RAS-inhibitors and statins. Pharmacies in arm B provided the intervention for RAS-inhibitors and statins and usual care for antidepressants and bisphosphonates.

**Intervention:** Intervention consisted of a telephone counseling intervention 7–21 days after the start of therapy. Counseling included assessment of practical and perceptual barriers and provision of information and motivation.

**Main outcome measure:** Primary outcome was refill adherence measured over 1 year expressed as continuous outcome and dichotomous (refill rate≥80%). Secondary outcome was discontinuation within 1 year.

**Results:** In the control arms 3627 patients were eligible and in the intervention arms 3094 patients. Of the latter, 1054 patients (34%) received the intervention. Intention to treat analysis showed no difference in adherence rates between the intervention and the usual care arm (74.7%, SD 37.5 respectively 74.5%, 37.9). More patients starting with RAS-inhibitors had a refill ratio ≥80% in the intervention arm compared to usual care (81.4 vs. 74.9% with odds ratio (OR) 1.43, 95%CI 1.11–1.99). Comparing patients with counseling to patients with usual care (per protocol analysis), adherence was statistically significant higher for patients starting with RAS-inhibitors, statins and bisphosphonates. Patients initiating antidepressants did not benefit from the intervention.

**Conclusions:** Telephone counseling at start of therapy improved adherence in patients initiating RAS-inhibitors. The per protocol analysis indicated an improvement for lipid lowering drugs and bisphosphonates. No effect for on adherence in patients initiating antidepressants was found.

The trial was registered at www.trialregister.nl under the identifier NTR3237.

## Introduction

Adherence to medication is a primary determinant of treatment success, and it is often suboptimal (Sabaté, 2003). Practical and perceptual barriers can prevent patients from adhering to the prescribed regimen. Practical barriers predominantly relate to cognition and self-efficacy whereas perceptual barriers predominantly relate to beliefs about the necessity and drawbacks of drug treatment (Linn et al., 2012). Health care providers (HCP) including pharmacists can reduce these barriers and thereby promote adherence (van Dulmen et al., 2007; Blom and Krass, 2011; van Hulten et al., 2011). Pharmacists have frequent interactions with patients, are easy accessible, well-trained and educated. Guidelines recommend counseling by pharmacists to improve medication adherence, especially at the start of therapy (1997; Puspitasari et al., 2009; Blom and Krass, 2011; van Hulten et al., 2011; Mendys et al., 2014). The first dispensing of a new drug, should be accompanied with general information and instructions for use. At the first refill, counseling should focus on exploring patients' experiences with the medication. In daily clinical practice, however guidelines are often not followed (Puspitasari et al., 2009; Van de Steeg-van Gompel et al., 2011; Schwappach et al., 2012) resulting in suboptimal counseling (Zolnierek and Dimatteo, 2009; Greenhill et al., 2011; Linn, 2013). The reasons for deviating from guidelines by pharmacists can be patient related (e.g., the patient is unable to visit the pharmacy, there are language problems, or the patient has low health literacy skills). Reasons can also be pharmacy or health system related (e.g., the pharmacy is understaffed, there is no priority for counseling, there is a lack of privacy or lack of remuneration). Telephone counseling may be a feasible alternative for face-to-face counseling (Elliott et al., 2008; Feifer et al., 2010). It has several advantages: first of all patient may be more comfortable when approached in their own environment where (lack) of privacy is not an issue. Moreover patients who are not able to visit the pharmacy can be reached. Also, pharmacists can prepare themselves on the call and the telephone calls can be planned (if necessary outside office hours). To test the effect of counseling by telephone on adherence, we designed a cluster randomized trial. Clusters (pharmacies) were randomized to increase the feasibility of including patients at the pharmacy counter. Primary goal of this study was to assess the effect of a pharmacist telephone counseling intervention on patients' medication adherence.

## Methods

### Study design

**Figure 1:**
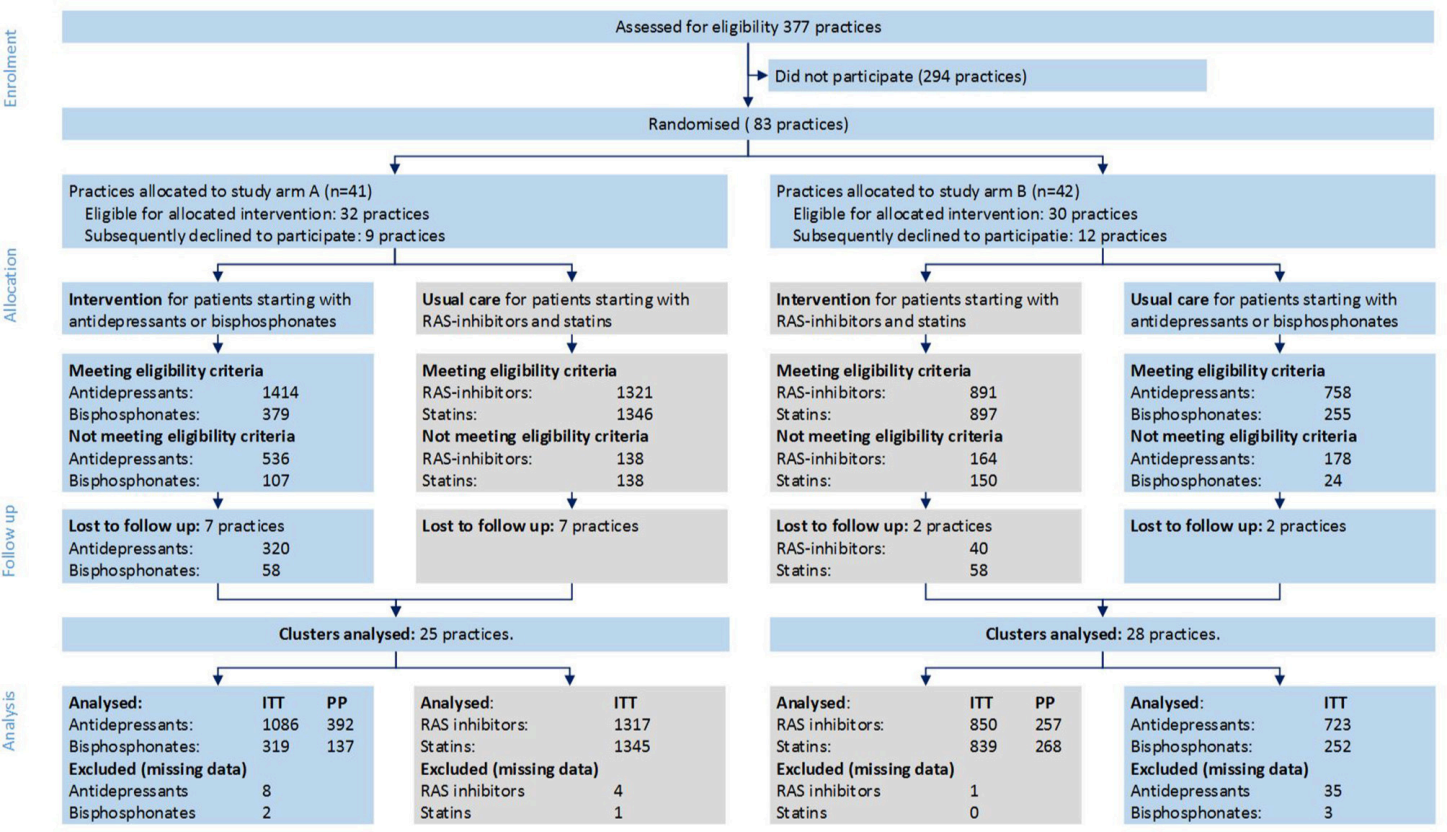
**Practice and patients flowchart**. This figure outlines the progress of clusters (pharmacies) and individual patients through the phases of the trial. Numbers are patients unless stated otherwise.

The protocol for this multicentre, community pharmacy based, cluster randomized controlled trial (the TelCIP trial) has been described elsewhere (Kooy et al., 2014). Pharmacies were randomized in two arms in a 1:1 ratio. Patients in arm A starting with antidepressants or bisphosphonates and patients in arm B starting with RAS-inhibitors or statins received the intervention. Patients in arm A starting with RAS-inhibitors or statins and patients in arm B starting with antidepressants or bisphosphonates received usual care (see Figure 1).

### Participants

#### Pharmacies

The study was conducted in community pharmacies in the Netherlands, both in rural and urban areas. No specific eligibility criteria for clusters were defined. Within every pharmacy one dedicated staff member was assigned to perform the intervention. This could be the pharmacist (PharmD) or a non-pharmacist employee with a bachelor degree (B), pharmacist trainee (BPharm) or pharmacy technician. In this paper we used the term pharmacist to describe all these staff members unless stated otherwise. The term pharmacy practitioner was used for bachelors and trainees. Pharmacists received a 3 h training aimed at understanding beliefs and behavior of patients related to medication intake. The training included case studies and an assessment of the level of theoretical knowledge on communication (Kooy et al., 2014). Pharmacist trainees did not receive additional training since communication is a currently taught throughout the curriculum (Blom et al., 2011, 2014).

#### Patients

Inclusion criteria were:

- Aged ≥ 18 years
- Speaking Dutch or same language as pharmacist/pharmacy worker
- Receiving a prescription for one of the fore mentioned medication classes for the first time in 12 months (Kooy et al., 2014).

Patients were excluded if the medication was prescribed for a short-term indication (e.g., antidepressant for smoking cessation or sleeping disorders), had a severe mental illness or were not responsible for their own drug intake. Patients were recruited between October 2010 and March 2013.

### Procedure

#### Usual care

Professional guidelines emphasize that patients receive both written and oral information at the start of pharmacotherapy. In practice this is mostly done by a pharmacy technician. The first dispensing provides medication for a maximum of 2 weeks. Guidelines recommend that at the first refill, patients are asked about their experiences with the medication. If necessary, additional information or counseling should be provided.

#### Intervention arm

Patients in the intervention arms were selected weekly through an automated selection procedure. Subsequently the pharmacist contacted patients by telephone between 7 and 21 days after the first prescription. Main goal of this call was to improve adherence. The call was supported by a pre-tested interview protocol aimed at addressing the following subjects: (1) need for information; (2) actual medication intake behavior; (3) practical barriers including side effects; (4) perceptual barriers including concerns or low necessity beliefs. The protocol was medication class specific and items like specific side effects and intake advices were included. A general interview protocol has been published elsewhere, together with the study protocol (Kooy et al., 2014).

#### Data collection

Pharmacy dispensing data were collected covering all prescriptions from at least 12 months before and after the intervention. These data included dispensing date, quantity dispensed, prescribed daily dose and prescriber.

### Outcome measures

The primary outcome was patients' refill adherence [Medication Possession Ratio modified, MPRm (Hess et al., 2006)] in the year following start of medication therapy, and was expressed both as a continuous and as a dichotomous measure; patients with an MPRm≥80% were considered adherent. The MPRm was calculated by dividing the total number of days' supply of a drug, excluding the last supply, by the number of days between the first and the last dispensing or first discontinuation date within the year after the start, whichever came first. The number of days supplied was calculated by dividing quantity dispensed by the prescribed daily dose. In case of missing dosing instructions, the instruction of the previous dispensing or the next dispensing (in case of a first prescription) was used. Retrospective compensation was not allowed.

Secondary outcome was persistence, which was expressed as the time from initiation until discontinuation. Discontinuation was defined as exceeding a gap of 90 days with no medication available within the 1-year observation period.

### Treatment fidelity

Treatment fidelity was promoted through the design of the study, by providing training to pharmacists, by providing a manual, an interview protocol and an online self-report (Borrelli, 2011). The self-report contained all items from the interview protocol and was used for continuously monitoring of implementation and for assessment of treatment fidelity. The pharmacists registered information on duration of the calls and number of attempts as well as the topics discussed and additional interventions performed. Reasons for not including a selected patient were also registered.

### Sample size

Sample size calculation was based on the primary outcome, the proportion of adherent patients (MPRm≥80%), using a type one error (α) for a two sided test of 0.05 and a power of 0.80 (1-β). To demonstrate an improvement of the proportion of adherent patients from 70 to 80%, an individually randomized trial would need 294 patients per arm and per medication class (Campbell et al., 1995). Correcting for clustering effects using an intracluster (or intraclass) correlation coefficient (ICC) of 0.02 resulted in the inclusion of at least 15 pharmacies with at least 30 patients each (Campbell et al., 1995; Eccles et al., 2003; Evans et al., 2009, 2010; Kooy et al., 2014).

### Data analysis

The primary analysis was based on the intention to treat (ITT) principle and the four medication classes were pre-defined subgroups. In a secondary, per protocol (PP) analysis, we compared patients in the intervention arm who actually received counseling, to patients in the usual care arm. Effect analyses were performed by a statistician (SB) blinded to the group allocation. Linear mixed-effects models were used for continuous outcomes and generalized mixed-effect models with the logit link function were used for dichotomous outcomes, both with pharmacy as random effect and percentile bootstrap confidence intervals with 1000 replications. Discontinuation in the first year was assessed using the Cox proportional hazards frailty model with the pharmacy as a random frailty factor. We considered a *p* < 0.05 to be statistically significant. For the descriptive and effect analyses we used R software version 3.1.2. (Austria, www.R-project.org). For multilevel analysis, library “lme4” was used with “lmer” function for continuous outcomes, “glmer” function for dichotomous outcomes and “survival” function for Cox regression. In a secondary, exploratory analysis we tested several factors as potential modifying factors: age, gender, Chronic Disease Score (CDS), and the status score at baseline. The CDS uses medication dispensed, as a surrogate marker for chronic illness (Von Korff et al., 1992). The status score (SS) is used as a marker for the individual socioeconomic status (SES). The SS is based on the patient's postal code and uses the average income, income, education and employment of persons living in that area (The statusscore presented by The Netherlands Institute for Social Research, 2014).

### Ethics and confidentiality

The Medical Ethics Review Committee (METC) of the University Medical Centre Utrecht has considered our research proposal in a meeting on 13 July 2010 and concluded that the Dutch Medical Research Involving Human Subjects Act (WMO) was not applicable. Consequently the protocol was submitted to the departmental Institutional Review Board (IRB) which approved the study protocol. The trial was registered at www.trialregister.nl under the identifier NTR3237. Patients received an information letter and gave informed consent before participating. All patient data were anonymised at the pharmacies.

## Results

Of 62 pharmacies that included patients in the study, dispensing data were available from 53 pharmacies (25 arm A and 28 arm B) (see Figure 1). In total 6731 patients were eligible (3627 control patients and 3094 intervention patients). A telephone call was registered for 1054 (34%) of the 3094 patients in the intervention arm. For 545 (18%) patients it was registered that the patient did not receive the intervention and for 1495 (48%) patients no registration was found.

Overall, patients in the intervention arm were younger and more often female (Table 1). However this was mainly due to the slight unequal distribution of medication classes over both arms. In the appendix additional information is provided: health characteristics are presented in Table A1, information at cluster level in Table A2 and on eligible patients without counseling in Table A3.

**Table 1:** **Baseline socio-demographic and health characteristics for each group at individual level**.

**Characteristic**	**Usual care**	**Eligible patients (ITT)**	**Patients with counseling (PP)**
**Overall**	*n* = 3637	*n* = 3094	*n* = 1054
Mean *(SD)* age, years	59.0 (15.1)	56.9 (15.9)	58.6 (15.8)
Female, *n* (%)	1987 (54.6)	1785 (57.7)	644 (61.1)
Mean *(SD)* status score	−0.44 (1.29)	−0.31 (1.20)	−0.43 (1.27)
Mean *(SD)* CDS	3.3 (3.1)	3.1 (3.1)	3.4 (3.2)
**Patients starting with RAS-inhibitor**	*n* = 1317	*n* = 850	*n* = 257
Mean *(SD)* age, years	61.1 (13.7)	62.2 (13.0)	63.8 (12.2)
Female, *n* (%)	710 (53.9)	439 (51.6)	145 (56.4)
Mean *(SD)* status score	−0.62 (1.32)	−0.01 (1.06)	−0.08 (1.18)
Mean *(SD)* CDS	3.3 (3.1)	3.3 (3.0)	3.5 (2.9)
**Patients starting with statin**	*n* = 1345	*n* = 839	*n* = 268
Mean *(SD)* age, years	60.6 (12.6)	61.6 (11.5)	62.5 (11.3)
Female, *n* (%)	660 (49.1)	414 (49.3)	139 (51.9)
Mean *(SD)* status score	−0.60 (1.29)	−0.02 (0.97)	−0.01 (0.98)
Mean *(SD)* CDS	3.4 (2.9)	3.4 (2.8)	3.6 (2.8)
**Patients starting with bisphosphonate**	*n* = 252	*n* = 319	*n* = 137
Mean *(SD)* age, years	66.5 (13.4)	66.2 (13.5)	67.8 (12.1)
Female, *n* (%)	186 (73.8)	251 (78.7)	111 (81.0)
Mean *(SD)* status score	0.14 (1.12)	−0.54 (1.25)	−0.64 (1.23)
Mean *(SD)* CDS	4.8 (3.9)	5.1 (3.7)	5.4 (3.7)
**Patients starting with antidepressant**	*n* = 723	*n* = 1086	*n* = 392
Mean *(SD)* age, years	49.4 (17.9)	46.5 (16.1)	49.3 (17.0)
Female	431 (59.6)	681 (62.7)	249 (63.5)
Mean *(SD)* status score	−0.03 (1.15)	−0.70 (1.33)	−0.89 (1.34)
Mean *(SD)* CDS	2.3 (2.9)	2.2 (2.9)	2.4 (3.1)

Values are numbers (percentages) unless stated otherwise.

In a secondary analysis we compared baseline characteristics for patients with counseling (PP) to patients in the usual care arm. Patients with counseling starting with RAS-inhibitors (*p* = 0.049) or statins (*p* = 0.04) where slightly older compared to patients with usual care. Other characteristics were not significantly different.

The most important reasons for not delivering the intervention were: no telephone number available (186, 32%), patient could not be reached (185, 31%), was not interested (83, 14%), or refused cooperation (44, 7%). On average the call lasted 8.3 min [Standard Deviation (*SD*) 4.4] and the average preparation time was 6.2 min (4.7). The pharmacists (PharmD) were responsible for 36% of the calls, pharmacy practitioners for 43% and technicians for 17%. In 79.5% of the calls all five knowledge items were reported to be discussed. These items included knowledge about reason of use (indication), mechanism of action, duration of treatment, correct moment of intake and possible side effects.

Doubts about necessity were discussed in 93.1% of the calls, concerns about side effects in 91.5% and experiences with side effects in 94.8%. According to the pharmacists 31.0% of the patients experienced side effects. In patients starting with antidepressants this proportion was higher compared to other medication classes (χ^2^-test *p* < 0.005).

### Primary outcome measures

#### Overall

In the overall ITT analysis we found a mean adherence rate (MPRm) of 74.7% (*SD* 37.5) for intervention patients and 74.5% (*SD* 37.9) for control patients (see Table 2). The proportion adherent patients (MPRm≥80%) was 69.0% in the intervention arm and 69.9% in the usual care arm and differences between intervention and usual care arms were not significantly different on both outcomes. Patients with counseling (PP-analysis) were not significantly more adherent (78.5% respectively 74.3%, see Table 2). However, when adjusted for age, gender, medication class and status score, the adjusted model demonstrated statistically higher adherence (5.79% 95%CI 2.57, 8.68) and significant more adherent patients in the intervention arm with an odds ratio of 1.48 (95% CI 1.20, 1.78).

**Table 2:** **Effect of telephone counseling on adherence expressed as mean adherence rate, proportion of adherent patients and discontinuation**.

**Variable**	**Usual care**	**Intervention arm (ITT)**	**Patients with counseling (PP)**	**ITT-analysis**	**PP-analysis**
				Effect size^*^ (95% CI)	Effect size^*^ (95% CI)
**Overall**	*n* = 3637	*n* = 3094	*n* = 1054		
Mean adherence *(SD)*	74.5 (37.9)	74.7 (37.5)	78.5 (35.4)	−1.07 (−8.13, 4.15)	3.96 (−2.34, 10.3)
Adherence ≥80%, % *(n)*	69.9 (2.519)	69.0 (2.134)	74.3 (783)	0.92 (0.68, 1.27)	1.30 (0.93, 2.00)
Discontinued, % *(n)*	33.2 (1.208)	34.6 (1.069)	33.4 (352)	1.08 (0.82, 1.37)	0.96 (0.70, 1.21)
**RAS**−**inhibitor users**	*n* = 1317	*n* = 850	*n* = 257		
Mean adherence *(SD)*	78.5 (36.6)	84.1 (31.6)	87.6 (26.4)	**5.16 (1.17, 10.03)**	**8.44 (2.01, 13.4)**
Adherence ≥80%, % *(n*)	74.9 (987)	81.4 (692)	84.1 (216)	**1.43 (1.11, 1.99)**	**1.71 (1.11, 2.62)**
Discontinued, % *(n)*	27.9 (367)	22.6 (192)	21.8 (56)	**0.77 (0.69, 0.91)**	0.73 (0.56, 1.02)
**Statin users**	*n* = 1345	*n* = 839	*n* = 268		
Mean adherence *(SD)*	75.1 (36.8)	80.5 (32.4)	85.2 (29.0)	4.08 (−0.81, 6.62)	**8.97 (3.51, 12.1)**
Adherence ≥80%, % *(n)*	68.9 (926)	75.1 (630)	81.3 (218)	1.27 (0.86, 1.54)	**1.83 (1.16, 2.49)**
Discontinued, % *(n)*	32.2 (433)	28.4 (238)	28.0 (75)	0.87 **(**0.73, 1.10)	0.82 (0.65,1.15)
**Bisphosphonate users**	*n* = 252)	*n* = 319	*n* = 137		
Mean adherence *(SD)*	73.3 (38.1)	75.2 (38.4)	84.3 (31.7)	−0.54 (−9.43,6.14)	**10.2 (1.98, 16.4)**
Adherence ≥80%, % *(n)*	67.1 (169)	70.2 (224)	81.8 (112)	1.00 (0.57, 1.49)	**2.15 (1.32, 3.57)**
Discontinued, % *(n)*	39.3 (99)	38.6 (123)	32.9 (45)	1.00 **(**0.80, 1.40)	0.79 (0.57, 1.25)
**Antidepressant users**	*n* = 723	*n* = 1086	*n* = 392		
Mean adherence *(SD)*	66.8 (40.9)	62.7 (41.7)	65.8 (41.7)	−3.78 (−8.15,0.93)	−0.55 (−6.04, 6.47)
Adherence ≥80%, % *(n)*	60.4 (437)	54.1 (588)	60.5 (237)	0.78 (0.59, 1.02)	1.05 (0.78, 1.58)
Discontinued, % *(n)*	42.7 (309)	47.5 (516)	44.9 (176)	**1.17 (1.01, 1.37)**	1.04 (0.84, 1.27)

* For “Mean adherence” the effect size is the “risk difference” and for proportion of for “Adherence ≥80%,” it is the Odds Ratio. The likelihood of being adherent is bigger (OR > 1) or smaller (OR < 1) for participants in the intervention arm compared with participants in the usual care arm. For discontinuation the effect size is the hazard ratio and the hazard of discontinuing in the first year is bigger (HR>1) or smaller (HR < 1) for participants in the intervention arm, compared with participants in the usual care arm. All presented CI's are bootstrap CI's. ITT, intention-to-treat; PP, per protocol; RAS, renin angiotensin system; CI, confidence interval; NNT, number needed to treat.

Bold: ES is outside 95% CI.

#### Effect per medication class

The mean adherence rate in patients in the intervention arm starting with RAS-inhibitors was 84.1% (SD 31.6) compared to 78.5% (SD 36.6) in the usual care arm which is a significant improvement with a adherence difference based on mixed-effect models of 5.16% (95% CI 1.17, 10.03) (See Table 2). In the intervention arm more patients were adherent (MPRm≥80%) compared to the usual care arm (81.4 vs. 74.9% with OR 1.43, 95% CI 1.11, 1.99). Effects on both outcomes were stronger and statistically significant for patients with counseling (PP-analysis). Based on the PP-analysis 16 patients need to be called in order for one extra patient to be adherent (NNT).

In statin users, patients in the intervention arm had a mean adherence rate of 80.5% (32.4) compared to 75.1% (36.8) in the usual care arm, which is a non-significant adherence difference of 4.08% (95% CI −0.81, 6.62). The proportion adherent patients in the intervention arm (75.1%) was not significantly different from the proportion in the usual care arm (68.9%) (OR 1.27, 95%CI 0.86, 1.54). Effects on both outcomes for patients with counseling (PP-analysis) were stronger and statistically significant. The number needed to call is 14.

In patients starting with bisphosphonates, the mean adherence rate in intervention arm (75.2%) was not different from the usual care arm (73.3%) neither was the proportion of adherent patients (70.2% respectively 67.1%). Effects on both outcomes for patients with counseling (PP-analysis) were stronger and statistically significant. The number needed to call is 11.

For antidepressants we found no significant difference in adherence rate between patients in the intervention arm (62.7% SD 41.7) and patients with usual care (66.8%, 40.9). The proportion adherent patients was also not significantly different between the arms (54.1% respectively 60.4% with OR 0.78, 95%CI 0.59, 1.02). In the PP-analysis we found no significant difference between arms.

Intracluster correlation coefficients are presented in Table A4.

### Secondary outcome measures

In the overall population we found no significant effect of the intervention on discontinuation in the first year after initiation (see Table 2). Also in the crude PP-analysis no statistically significant difference was found but in the adjusted model discontinuation was lower [hazard ratio (HR) 0.87, 95% CI 0.80, 0.95]. For patients starting with RAS-inhibitors 22.6% of the patients in the intervention arm discontinued therapy compared to 27.9% of patients with usual care which is significantly lower (HR 0.77, 95% CI 0.69, 0.91). The result of the PP-analysis is comparable but not statistically significant. For statins and bisphosphonate users, discontinuation rates were not significantly different (HR 0.87, 95% CI 0.73, 1.10, respectively 0.73, 95% CI 0.56, 1.02).

For patients starting with antidepressants in the intervention arm 47.5% discontinued compared to 42.7% in the usual care arm which is a significant different (HR 1.17, 95% CI 1.01, 1.37). However in the PP-analysis 44.9% discontinued in the intervention arm which is not significantly different from usual care (HR 1.04, 95%CI 0.84, 1.27)

## Discussion

The aim of this study was to assess the effect of telephone counseling at start of therapy on adherence to RAS-inhibitors, statins, bisphosphonates, and antidepressants. Overall, no effect of the intervention was found. Results suggest that adherence improved for patients starting with RAS-inhibitors, statins or bisphosphonates. For antidepressants no significant effect was found.

An interim analysis of this study already showed that telephone counseling increased satisfaction with information and increased satisfaction with counseling (Kooy et al., 2015). The current analysis demonstrates that this increased satisfaction may translate into improved medication adherence for some medication classes.

Baseline characteristics of non-registered patients were not different from patients with counseling. However to prevent bias, we included all eligible patients based on the prescription data in the ITT analysis which diluted the potential effect of the intervention. In line with the expectations, effects in the PP-analysis were stronger for most outcomes.

The lack of improvement of adherence to antidepressant therapy is in line with other published studies (Chong et al., 2013). In a review of interventions focussing on antidepressants (Chong et al., 2011) authors suggest that educational intervention alone is not enough and that complex interventions are needed. However, a recent review indicated that pharmacist care can improve adherence to antidepressants (Rubio-Valera et al., 2011). In all studies included in this review, patients had a verified diagnosis of depression. This is missing in our study and it is unknown if the medication was prescribed for depression or other indications like anxiety disorders. Based on the dispensing data however we know that < 20% of patients received a prescription from a psychiatrist in the 12 months before the start of the antidepressant. Our study showed that patients using antidepressants were more likely to experience side effects. This may have been an additional barrier to improve adherence. Moreover the counseling might have helped patients to make a thought-out decision whether or not to continue treatment, although the effect on adherence might be the same, (Kooy et al., 2015) Studies for other medication classes like antidiabetics, (Sacco et al., 2009; Walker et al., 2011), antiplatelet medication (Rinfret et al., 2013), and statins (Derose et al., 2013) showed a positive effect of counseling on adherence or clinical outcomes. In literature we found no trials with a comparable intervention studied for antihypertensives. In a trial focussing on bisphosphonates no statistically significant improvement of adherence was found using a telephone motivational interviewing intervention (Solomon et al., 2012).

Mean adherence rates and proportion of adherent patients were relatively high in our study population, both in the intervention as in the usual care arm. One explanation might be that participating pharmacies have already implemented counseling guidelines to a large extent. Comparison of adherence between studies should, however, be performed with caution as the calculated refill-rate is influenced by the method of calculation and assumptions made (Hess et al., 2006).

Strength of this study is that it was implemented in a real-life setting and with four different medication classes. The pragmatic design of the trial and the inclusion of a large number of pharmacies contributes to the generalizability of the results. Another strength is that a relatively high proportion of eligible patients received the intervention. This is high compared to what is known from literature (Puspitasari et al., 2009; Van de Steeg-van Gompel et al., 2011; Boeni et al., 2015; Dijk et al., 2015). Moreover this intervention includes patients irrespective if they return for a refill or not. So also patients who decided not to initiate or to discontinue were approached. This is relevant since a substantial part of the patients discontinue therapy in the first weeks. Patients not capable of visiting the pharmacy were also included. Different strategies were used to enhance treatment fidelity for example by preventing contamination using a cluster design, providing standardized training, providing medication class specific interview protocols and treatment manual, and the obligation for pharmacists to complete a self-report questionnaire for every selected patient. Moreover pharmacies received biweekly updates of the number of patients included with benchmark information. The intervention was based on theoretical models and guidelines and the interview protocol contained pre-defined questions and relevant knowledge items. Moreover the protocol stimulated pharmacists to ask about patient opinions, for example by asking “What do you think of getting this medicine?” and to tailor counseling to patients' needs which is important to improve adherence (Andersson et al., 2014). Our study also has its limitations. Based on the prescription data, more patients should have received the intervention than were registered. The exact reason is not known but possible explanations can be that (1) the computer program did not select all patients, (2) the pharmacist did not run the program or temporarily stopped including patients, (3) the pharmacist did call the patient, but failed to register it, and (4) the pharmacist decided not to call the patient for unknown reasons. During interviews with some pharmacists they indicated to have (temporarily) stopped including patients due to staffing problems.

Patients have different needs and variability between HCP in the care they provide exists. More research is needed to identify for which patients and in which setting standard care is sufficient and for which patients standard care is not sufficient and thus need additional counseling for example by telephone. Moreover the cost-effectiveness of the intervention needs to be assessed.

### Conclusion

Telephone counseling at start of therapy improved adherence in patients initiating RAS-inhibitors. The per protocol analysis indicated an improvement for lipid lowering drugs and bisphosphonates. No effect for on adherence in patients initiating antidepressants was found.

## Author contributions

MK wrote the first draft of the manuscript. MK, MB, EV, SB, and EH participated in the design of the trial and study methodology. SB performed the analysis. MB, EV, SB, EH, and LV reviewed the manuscript and made critical revisions. All authors read and approved the final manuscript.

## Funding and disclosure

The Royal Dutch Pharmacists Association (KNMP) provided financial support of the study and pharmacists received remuneration from a health insurance company (Zilveren Kruis Achmea). All authors have completed the ICMJE uniform disclosure form at www.icmje.org/coi_disclosure.pdf and declare: Dr. MB reports grants from Royal Dutch Pharmacists Association (KNMP), during the conduct of the study; no financial relationships with any organizations that might have an interest in the submitted work in the previous 3 years; no other relationships or activities that could appear to have influenced the submitted work. Dr. LV reports grants from Astra Zeneca, grants from Pfizer, grants from Bristol-Myer Squib, outside the submitted work.

### Conflict of interest statement

The authors declare that the research was conducted in the absence of any commercial or financial relationships that could be construed as a potential conflict of interest.

## Appendix

**Table A1:** **Baseline health characteristics for each group at patient level**.

**Characteristic**	**Usual care**	**Eligible patients (ITT)**	**Patients with counseling (PP)**	**Overall**
**USE OF MEDICATION PRECEDING INDEX DATE:**				
**Patients starting with RAS-inhibitor**	*n* = 1317	*n* = 850	*n* = 257	*n* = 2167
Antidiabetics (A10)	176 (13.3)	96 (11.2)	36 (14.0)	272 (12.6)
Antithrombotics (B01)	295 (22.3)	227 (26.7)	67 (26.1)	522 (24.1)
Antihypertensives	604 (46.8)	438 (52.5)	134 (53.0)	1042 (49.1)
**Patients starting with statin**	*n* = 1345	*n* = 839	*n* = 268	*n* = 2184
Antidiabetics (A10)	246 (18.3)	128 (15.3)	34 (12.7)	374 (17.1)
Antithrombotics (B01)	425 (31.6)	279 (33.3)	79 (29.5)	704 (32.2)
Antihypertensives	526 (39.3)	369 (44.5)	126 (47.5)	895 (41.3)
Visit to cardiologist or internist in 12 months before index	465 (34.6)	280 (33.3)	90 (33.6)	745 (34.1)
**Patients starting with bisphosphonate**	*n* = 252	*n* = 319	*n* = 137	*n* = 571
Calcium suppletion (A12A)	123 (48.8)	163 (51.1)	68 (49.6)	286 (50.1)
Predniso(lo)ne (H02AB06 or H02AB07)	68 (27.0)	85 (26.6)	38 (27.7)	153 (26.8)
Vitamin D (A11CC05)	115 (45.6)	155 (48.6)	67 (48.9)	270 (47.3)
Visit to internist in 12 months before index	248 (98.4)	298 (93.4)	132 (96.4)	546 (95.6)
**Patients starting with antidepressant**	*n* = 723	*n* = 1086	*n* = 392	*n* = 1809
Antipsychotic (N05)	47 (6.5)	53 (4.9)	12 (3.1)	100 (5.5)
Benzodiazepin (N05BA, N05CD or N05CF)	250 (34.6)	307 (28.3)	109 (27.9)	557 (30.8)
Visit to psychiatrist in 12 months before index	141 (19.5)	276 (25.4)	99 (25.3)	417 (23.1)

Values are number (percentages) unless stated otherwise.

Antihypertensives: Antihypertensives (C02) + Diuretics (C03) + Beta blocking agents (BBA, C07) + Calcium channel blockers (CCB, C08) + RAS inhibitors (C09)).

**Table A2:** **Baseline socio-demographic and health characteristics for each group at cluster level**.

**Characteristic**	**Usual care**	**Eligible patients (ITT)**	**Patients with counseling (PP)**	**Overall**
**GENDER (NO/CLUSTER)**				
Female	38.2 (34.3)	34.3 (41.2)	12.6 (17.6)	72.5 (85.7)
Male	31.7 (44.1)	25.2 (30.6)	8.0 (10.0)	56.9 (64.0)
**PHARMACY INCLUSION PHASE**				
Phase 1	113.9 (102.5)	95.2 (76.3)	33.4 (29.8)	209.2 (158.3)
Phase 2	14.5 (14.4)	14.4 (13.7)	3.8 (2.8)	28.9 (24.4)
**AGE GROUPS (NO/CLUSTER)**				
18–50	19.7 (22.5)	20.3 (28.2)	6.4 (11.5)	40.0 (49.3)
51–65	25.8 (37.4)	19.8 (25.4)	6.6 (8.6)	45.6 (52.1)
>65	24.5 (35.5)	19.4 (26.9)	7.6 (11.5)	43.8 (51.4)
**NUMBER OF PATIENTS STARTING**				
Overall	69.9 (91.2)	59.5 (70.1)	20.7 (26.8)	129.4 (149.0)
RAS-inhibitors	25.3 (48.1)	16.3 (31.0)	9.5 (10.7)	41.7 (49.3)
Statins	25.9 (48.7)	16.1 (33.5)	9.9 (14.0)	42.0 (51.4)
Bisphosphonates	4.8 (8.1)	6.1 (11.4)	5.7 (8.2)	11.0 (11.6)
Antidepressants	13.9 (23.3)	20.9 (40.5)	16.3 (23.4)	34.8 (39.9)
**CHRONIC DISEASE SCORE**				
0–1	27.3 (33.0)	24.8 (29.1)	7.9 (11.5)	52.0 (59.9)
2–4	27.3 (33.0)	16.9 (22.7)	5.8 (8.3)	38.4 (43.8)
5–19	21.2 (29.5)	16.5 (21.2)	6.4 (8.0)	37.7 (45.0)
Status score				
< –1.0	24.4 (55.4)	20.5 (45.1)	7.7 (18.2)	44.9 (96.1)
–1 to 0.5	24.4 (44.3)	18.0 (29.5)	6.2 (12.2)	42.4 (67.6)
>0.5	21.1 (33.6)	21.1 (35.0)	6.7 (10.7)	42.2 (64.2)

Values are means per cluster (standard deviation) unless stated otherwise.

**Table A3:** **Baseline socio-demographic and health characteristics for each group at individual level**.

**Characteristic**	**Usual care**	**Eligible patients (ITT)**	**Patients with counseling (PP)**	**Eligible patients without counseling**	**Overall**
**Overall**	*n* = 3637	*n* = 3094	*n* = 1054	*n* = 2040	*n* = 6731
Mean *(SD)* age, years	59.0 (15.1)	56.9 (15.9)	58.6 (15.8)	58.0 (15.5)	56.1(15.9)
Female, *n* (%)	1987 (54.6)	1785 (57.7)	644 (61.1)	3772 (56.0)	1141 (55.9)
Mean *(SD)* status score	−0.44 (1.29)	−0.31 (1.20)	−0.43 (1.27)	−0.38 (1.25)	−0.24(1.16)
Mean *(SD)* CDS	3.3 (3.1)	3.1 (3.1)	3.4 (3.2)	3.2 (3.1)	3.0(3.1)
**Starting with RAS-inhibitor**	*n* = 1317	*n* = 850	*n* = 257	*n* = 593	*n* = 2167
Mean *(SD)* age, years	61.1 (13.7)	62.2 (13.0)	63.8 (12.2)	61.6 (13.4)	61.5 (13.3)
Female, *n* (%)	710 (53.9)	439 (51.6)	145 (56.4)	1149 (53.0)	294 (49.6)
Mean *(SD)* status score	−0.62 (1.32)	−0.01 (1.06)	−0.08 (1.18)	−0.38 (1.26)	0.02 (1.0)
Mean *(SD)* CDS	3.3 (3.1)	3.3 (3.0)	3.5 (2.9)	3.3 (3.1)	3.2 (3.0)
**Starting with statin**	*n* = 1345	*n* = 839	*n* = 268	*n* = 571	*n* = 2184
Mean *(SD)* age, years	60.6 (12.6)	61.6 (11.5)	62.5 (11.3)	61.0 (12.2)	61.2 (11.5)
Female, *n* (%)	660 (49.1)	414 (49.3)	139 (51.9)	1074 (49.2)	275 (48.2)
Mean *(SD)* status score	−0.60 (1.29)	−0.02 (0.97)	−0.01 (0.98)	−0.38 (1.21)	−0.03 (0.97)
Mean *(SD)* CDS	3.4 (2.9)	3.4 (2.8)	3.6 (2.8)	3.4 (2.9)	3.3 (2.8)
**Starting with bisphosphonate**	*n* = 252	*n* = 319	*n* = 137	*n* = 182	*n* = 571
Mean *(SD)* age, years	66.5 (13.4)	66.2 (13.5)	67.8 (12.1)	66.3 (13.5)	64.9 (14.4)
Female, *n* (%)	186 (73.8)	251 (78.7)	111 (81.0)	437 (76.5)	140 (76.9)
Mean *(SD)* status score	0.14 (1.12)	−0.54 (1.25)	−0.64 (1.23)	−0.24 (1.24)	−0.46 (1.26)
Mean *(SD)* CDS	4.8 (3.9)	5.1 (3.7)	5.4 (3.7)	5.0 (3.8)	4.9 (3.7)
**Starting with antidepressant**	*n* = 723	*n* = 1086	*n* = 392	*n* = 694	*n* = 1086
Mean *(SD)* age, years	49.4 (17.9)	46.5 (16.1)	49.3 (17.0)	47.7 (16.9)	44.9 (15.4)
Female, *n* (%)	431 (59.6)	681 (62.7)	249 (63.5)	1112 (61.5)	432 (62.2)
Mean *(SD)* status score	−0.03 (1.15)	−0.70 (1.33)	−0.89 (1.34)	−0.43 (1.30)	−0.59 (1.31)
Mean *(SD)* CDS	2.3 (2.9)	2.2 (2.9)	2.4 (3.1)	2.3 (2.9)	2.0 (2.8)

Values are numbers (percentages) unless stated otherwise.

**Table A4:** **Coefficients of intracluster correlation (ICC)**.

**Medicationclass**	**Refillrate (MPRm)**	**Refillrate (PDC)**
**Overall**	0.022	0.020
RAS-inhibitors	0.032	0.022
Statins	0.011	0.004
Bisphosphonates	0.058	0.036
